# Proteome analysis of human amniotic mesenchymal stem cells (hA-MSCs) reveals impaired antioxidant ability, cytoskeleton and metabolic functionality in maternal obesity

**DOI:** 10.1038/srep25270

**Published:** 2016-04-29

**Authors:** Valentina Capobianco, Marianna Caterino, Laura Iaffaldano, Carmela Nardelli, Angelo Sirico, Luigi Del Vecchio, Pasquale Martinelli, Lucio Pastore, Pietro Pucci, Lucia Sacchetti

**Affiliations:** 1 CEINGE-Biotecnologie Avanzate S.C.a R.L., Via Gaetano Salvatore 486, 80145 Naples, Italy; 2 Dipartimento di Medicina Molecolare e Biotecnologie Mediche, Università degli Studi di Napoli Federico II, Via S. Pansini 5, 80131 Naples, Italy; 3 Dipartimento di Neuroscienze e Scienze Riproduttive ed Odontostomatologiche, Via S. Pansini 5, 80131 Naples, Italy; 4 Dipartimento di Scienze chimiche, Via Cintia, Complesso Monte Sant’Angelo 21, 80126 Naples, Italy

## Abstract

Maternal obesity increases the risk of obesity and/or obesity-related diseases in the offspring of animal models. The aim of this study was to identify metabolic dysfunctions that could represent an enhanced risk for human obesity or obesity-related diseases in newborn or in adult life, similar to what occurs in animal models. To this aim, we studied the proteome of 12 obese (Ob-) and 6 non-obese (Co-) human amniotic mesenchymal stem cells (hA-MSCs) obtained from women at delivery by cesarean section (pre-pregnancy body mass index [mean ± SD]: 42.7 ± 7.7 and 21.3 ± 3.3 kg/m^2^, respectively). The proteome, investigated by two-dimensional fluorescence difference gel electrophoresis/mass spectrometry, revealed 62 differently expressed proteins in Ob- vs Co-hA-MSCs (P < 0.05), nine of which were confirmed by western blotting. Bioinformatics analysis showed that these 62 proteins are involved in several statistically significant pathways (P < 0.05), including the stress response, cytoskeleton and metabolic pathways. Oxidative stress was shown to be an early triggering factor of tissue fat accumulation and obesity-related disorders in the offspring of obese animal models. Our finding of a reduced stress response in Ob-hA-MSCs suggests that a similar mechanism could occur also in humans. Long-term follow-up studies of newborns of obese mothers are required to verify this hypothesis.

Obesity is a worldwide epidemic health problem, and up to 60% of pregnant women are obese/overweight before pregnancy^1^. Clinical studies and findings obtained in animal models suggest that developmental programming in the presence of maternal obesity and nutrient excess *in utero* increases the risk of offspring to develop obesity and/or obesity-associated metabolic diseases later in life^2^,^3^. In humans, amnion from placental tissue is the main interface between the fetus and mother: it regulates intrauterine development and modulates adaptive responses to suboptimal *in utero* conditions such as obesity and/or an obesogenic diet^4^. Accordingly, during obesity, placental tissues undergo epigenetic and proteome alterations that involve pathways that are crucial for placental function and fetal growth^5^,^6^. Amnion is a source of fetal mesenchymal stem cells (hA-MSCs) that have a close ontogenic relationship with embryonic stem cells, and unlike the latter, they are accessible without ethical problems because the placenta is usually discarded at birth^7^. Furthermore, hA-MSCs have multipotent differentiation potential, including the potential to differentiate in adipocytes, and are thus a useful cellular model with which to investigate dysfunctions in adipose tissue during obesity^7^. In particular, studies on hA-MSCs could reveal the metabolic alterations that, if present at perinatal level, could impact on the fetal developmental program.

We recently reported that the adipogenic potential of hA-MSCs isolated from obese women (Ob-hA-MSCs) was higher than that of hA-MSCs from lean control women (Co-hA-MSCs)^8^. In detail, we demonstrated that high levels of CD13/aminopeptidase N on the Ob-hA-MSC surface, measured by immunophenotyping, resulted in enhanced adipogenesis of these cells, which, in turn, could be related to the pathogenesis of obesity^8^. Based on these encouraging results, we analyzed the entire proteome of Ob-hA-MSCs in the attempt to identify metabolic dysfunctions that, being present at perinatal level, could represent an enhanced risk for obesity or for obesity-related diseases in the newborn or in adult life, similar to what occurs in animal models^3^.

## Results

Several anamnestic and biochemical characteristics (Supplementary Table S1), recorded immediately before delivery, were similar in the two groups of enrolled pregnant women, whereas serum leptin (P = 0.002) and the L/A ratio (P = 0.01) were significantly higher, and serum adiponectin lower, albeit not significantly so, in obese women than in non-obese women. The mean (±SD) pre-pregnancy body mass index (BMI, kg/m^2^) was higher [42.7(7.7) vs 21.3(3.3), P < 0.0001], and the weight gain in pregnancy was lower (9.5 vs 14.0) in obese than in non-obese women, as recommended by guidelines^9^ (Supplementary Table S1). Birth weight and length, head circumference, and the 1 and 5 min Apgar indexes did not differ between Ob- and Co-newborns (Supplementary Table S1).

### Protein profile of hA-MSCs

**Figure 1:**
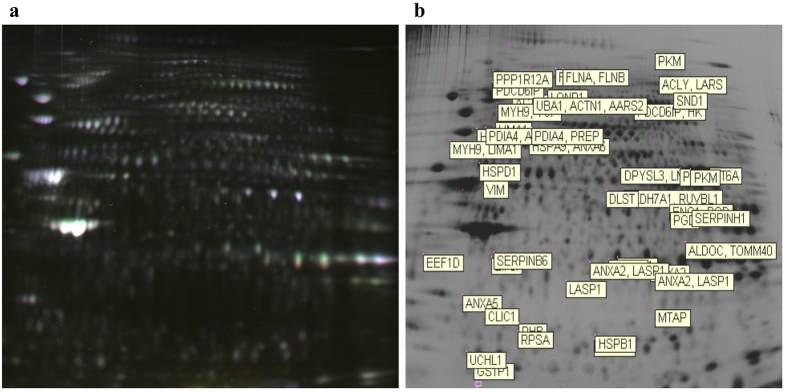
Two-Dimensional Fluorescence Difference Gel Electrophoresis of hA-MSC proteins. (**a)** Scan of the master gel used to match protein spots from the six analytical gels used for image analysis. This gel is constituted by three overlapping images: (1) proteins from obese hA-MSCs labeled with Cy3 (green); (2) proteins from non-obese hA-MSCs labeled with Cy5 (red); and (3) proteins from a pool of all samples labeled with Cy2 (blue) (used for normalization). (**b)** The NCBI gene symbol for each differently expressed protein is reported as indicated in Table 1.

The master gel used to match the whole set of two-dimensional fluorescence difference gel electrophoresis (2D-DIGE) profiles obtained in Ob-hA-MSCs and Co-hA-MSCs is shown in Fig. 1a,b. The analysis performed with DeCyder Software revealed approximately 7000 protein spots per gel in the 3–10 pH range. Approximately 2384 spots were matched in all six analytical gels. Spots at the extreme right and left sides of the gel were excluded from the evaluation. The DeCyder statistical analysis revealed 159 spots differentially expressed at a statistical significant level (P < 0.05), with an average ratio ≥1.2 for up-expressed and ≤−1.2 for down-expressed protein spots, in Ob-hA-MSCs versus Co-hA-MSCs. The preparative gel was stained with Comassie Brilliant-blue and used for automated spot picking. After excluding 43 spots for their low abundance, we focused on 116 spots that were very abundant on the analytical gel or on the preparative gel. The isolated spots were digested with trypsin and analyzed with mass spectrometry followed by database search. Sixty protein spots were considered informative.

**Figure 2:**
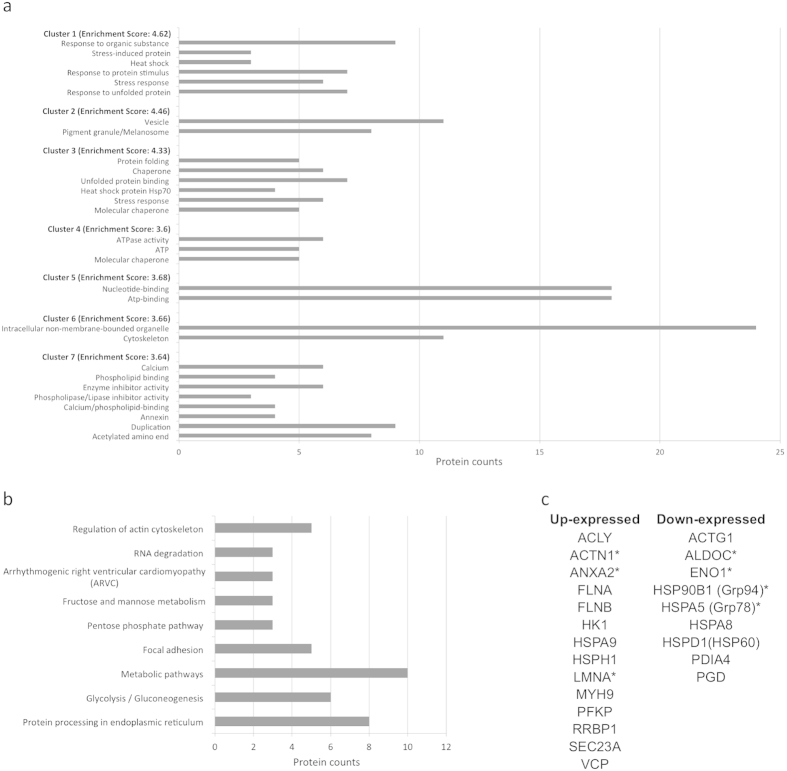
Pathways predicted by the DAVID and KEGG databases and list of proteins involved in pathways predicted by both tools. **(a)** Functional annotation clustering obtained with DAVID: cluster 1 (response to unfolded protein; stress response; response to protein stimulus; heat shock; stress-induced protein; response to organic substance); cluster 2 (pigment granule/melanosome; vesicle); cluster 3 (molecular chaperone; stress response; heat shock protein 70; unfolded protein binding; chaperone; protein folding); cluster 4 (molecular chaperone; ATP; ATPase activity); cluster 5 (ATP-binding; nucleotide-binding); cluster 6 (cytoskeleton; intracellular non-membrane-bounded organelle); and cluster 7 (acetylated amino end; duplication; annexin; calcium binding; phospholipase/lipase inhibitor activity; enzyme inhibitor activity; phospholipid binding; calcium). **(b)** Statistically significant pathways predicted by KEGG: protein processing in the endoplasmic reticulum; glycolysis/gluconeogenesis; metabolic pathways; focal adhesion; the pentose phosphate pathway; fructose and mannose metabolism; arrhythmogenic right ventricular cardiomyopathy; RNA degradation; regulation of actin cytoskeleton. Grey bar represent the protein counts, that is the number of proteins included in each pathway. **(c)** List of proteins involved in statistical significant pathways predicted by both tools. ^*^Proteins tested by Western Blot.

The relative expression ratios of each informative spot (Ob- vs Co-hA-MSCs), their statistical significant values (P < 0.05) and general characteristics of the proteins identified in each spot are listed in Table 1. Sixty-two per cent (37/60) of the spots were up-expressed, and 38% (23/60) were down-expressed. A total of 62 proteins was identified. We used the DAVID and KEGG databases to identify the statistically significant pathways (P < 0.001) containing the differentially expressed proteins (Supplementary Table S2). Among the pathways predicted by DAVID we arbitrarily considered only those grouped in highly scored clusters (4.62–3.64 enrichment score): cluster 1 (response to unfolded protein; stress response; response to protein stimulus; heat shock; stress-induced protein; response to organic substance); cluster 2 (pigment granule/melanosome; vesicle); cluster 3 (molecular chaperone; stress response; heat shock protein 70; unfolded protein binding; Chaperone; protein folding); cluster 4 (molecular chaperone; ATP; ATPase activity); cluster 5 (ATP-binding; nucleotide-binding); cluster 6 (cytoskeleton; intracellular non-membrane-bounded organelle); and cluster 7 (acetylated amino end; duplication; annexin; calcium binding; phospholipase/lipase inhibitor activity; enzyme inhibitor activity; phospholipid binding; calcium) (Fig. 2a). Figure 2b shows the statistical significant pathways (P < 0.05) predicted by KEGG: protein processing in the endoplasmic reticulum; glycolysis/gluconeogenesis; metabolic pathways; focal adhesion; the pentose phosphate pathway; fructose and mannose metabolism; arrhythmogenic right ventricular cardiomyopathy; RNA degradation; regulation of actin cytoskeleton. The proteins involved in statistical significant pathways predicted by both tools are listed in Fig. 2c.

**Figure 3:**
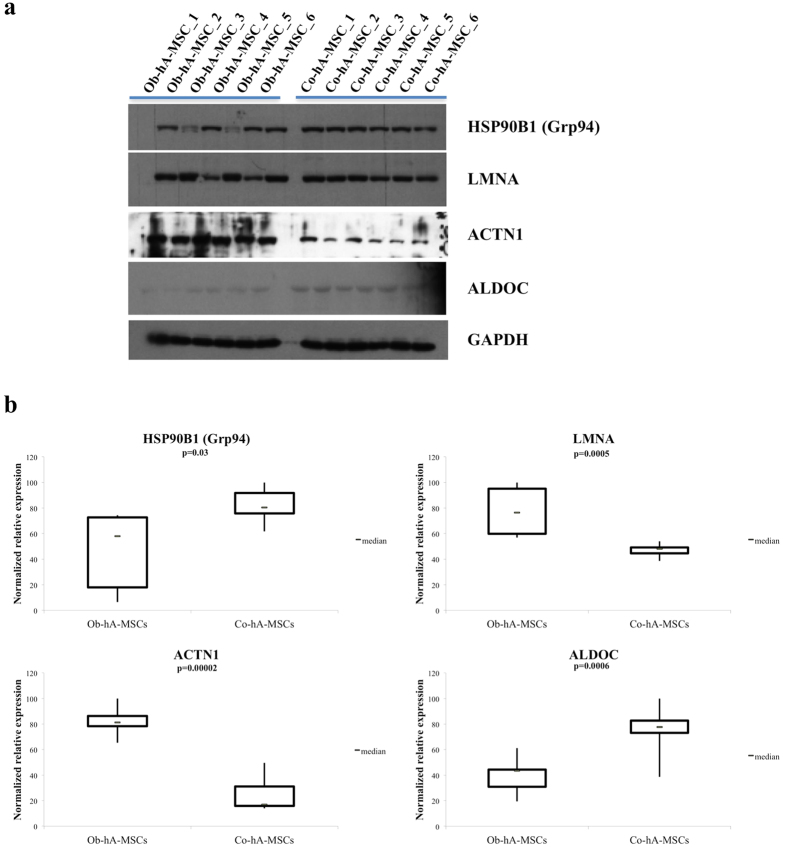
Western blot evaluation of selected proteins. **(a)** Western blot showing statistically significant different levels (P < 0.05) of four proteins (HSP90B1, LMNA, ACTN1 and ALDOC) in Ob-hA-MSCs vs Co-hA-MSCs. In the figure are reported the cropped gels/blots obtained by each protein evaluation. All gels were run in the same experimental conditions (see material and methods for details). (Full-length blots of each tested protein are reported in Supplementary Figure S2). (**b**) Quantification of the expression of the tested four proteins. The optical density of each sample was measured and normalized using a GAPDH run on the same gel. The data are expressed as percent relative expression, i.e. for each protein, the sample with the highest expression of that protein was set at 100%. The bottom and top of each box represent the 25th and 75th percentile, respectively; the thick band within each box shows the 50th percentile (the median). The ends of the whiskers represent the minimum and maximum values of each group of data.

To confirm the protein levels obtained at 2D-DIGE, we measured the expression of nine proteins [4/9 up-expressed: pyruvate kinase (PKM), alpha-actinin 1 (ACTN1), prelamin A/C (LMNA), annexin A2 (ANXA2)], and 5/9 down-expressed: endoplasmin (HSP90B1/Grp94), aldolase C (ALDOC), heat-shock protein beta-1 (HSPB1/HSP27), 79 kDa glucose-regulated protein (HSPA5/Grp78), enolase 1 (ENO1)] in Ob- versus Co-hA-MSCs], by western blot (WB). The proteins were selected by arbitrary selection criteria: 7/9 proteins predicted by both DAVID and KEGG tools, 1/9 of those predicted only by DAVID and 1/9 of those predicted only by KEGG. For all nine tested proteins, the expression levels obtained at WB analysis confirmed the trend obtained at 2D-DIGE, and the differences in the WB expression levels (mean/SEM) between Ob- and Co-hA-MSCs were statistically significant (P < 0.05) for 4/9 tested proteins: HSP90B1 (0.23/0.06 and 0.41/0.03), LMNA (0.86/0.23 and 0.52/0.05), ACTN1 (1.41/0.08 and 0.43/0.10) and ALDOC (0.62/0.09 and 1.16/0.13) (Fig. 3a,b; Supplementary Figure S1). Full-length blots of each tested protein are reported in Supplementary Figure S2.

## Discussion

In this study, we show that the proteome of hA-MSCs isolated from obese women at delivery differs significantly from that of control women. This finding, together with our previous report of enhanced adipogenesis in Ob-hA-MSCs and of an altered miRNA expression profile in amnion of obese women at delivery^5^,^8^, helps to shed light on the alterations that occur in the intrauterine environment in human obesity.

Globally, we identified, at 2D-DIGE followed by mass spectrometry, 62 proteins that were differently expressed in Ob- vs Co-hA-MSCs, and that were predicted by bioinformatics to alter many relevant cellular pathways. However, given the large number of proteins/pathways predicted to be deregulated, we focused our efforts on three groups of proteins that are involved in the stress response, the cytoskeleton and metabolism, respectively, that, based on findings obtained in pregnant obese animal models and their offspring^10^,^11^, and in tissues from adult obese subjects^6^, could be of significance in predisposing newborn of obese women to obesity or to obesity-related diseases.

Regarding the stress response, we found that a group of HSP proteins involved in this pathway (HSPA8, HSPD1, HSPB1, HSP90B1 and HSPA5) were down-expressed in Ob- vs Co-hA-MSCs. Heat shock proteins have been linked to obesity, inflammation and insulin resistance^12^. Heat shock proteins, which are stress-induced and mainly involved in cellular protein processing, are a group of highly conserved and ubiquitously expressed proteins discovered 50 years ago when, the accidental application of heat shock to *Drosophila* induced their expression^13^. HSPA8, a member of the HSP 70 family, is mainly expressed in the cytosol and nucleus, and represents up to 1% of the total cellular protein content^14^. HSPA8 binds to nascent or unfolded proteins in an ADP/ATP-dependent manner and exerts its chaperone activity often cooperating with other co-chaperones^14^. It is required for clathrin-mediated endocytosis, protein folding and protein degradation in the ubiquitin-proteasome pathway, and it plays a decisive role in autophagy by targeting proteins for lysosomal degradation^14^,^15^. HSPA8 is also endowed with shuttling capacity, being able to import/export proteins from the cytoplasm to the nucleus and *vice versa*, but this property can be inhibited by stress^16^, a condition in which HSPA8 is confined to the nucleus. Lastly, HSPA8 is involved in the mitochondrial import of proteins via its interaction with HSP90^17^.

Also HSP90B1 was decreased in our Ob-hA-MSCs. HSPD1, a mitochondrial chaperone of the HSP family, exerts a fundamental role in mitochondrial homeostasis and insulin sensitivity. In fact, in a murine model of type II diabetes mellitus, downregulation of mitochondrial HSPD1 caused hypothalamic insulin resistance and mitochondrial dysfunction consequent to disruption of leptin signaling^18^. HSPB1 is an ATP-independent molecular chaperone that enables the cell to respond to and overcome different stress conditions^19^. HSPA5 is located in the endoplasmic reticulum where it is involved in the import and folding of newly synthesized proteins^14^. The HSP90B1, HSPA5 and HSPD1 proteins have been implicated in inflammatory responses and stress response^20^.

Globally, the above five HSP proteins are involved in protecting cell integrity and in defending cells against oxidative stress. Consequently, their decreased levels in hA-MSCs in cases of maternal obesity is compatible with a defective response to stress in these cells. Maternal obesity exacerbates perinatal stress. In fact, placentas obtained from obese women show macrophage accumulation and inflammation similar to adipose tissue from obese subjects^21^. In animal models, maternal obesity results in the development of obesity-related diseases in offspring^10^. In fact, pancreatic and liver oxidative stress and apoptosis markers were significantly higher both in fetal tissue and in adult offspring of 25-week-old obese dams than in offspring from control dams, irrespective of their diet, and the increase was maintained up to the age of 30 weeks^10^. Moreover, metabolic alterations were associated with mitochondrial dysfunction and reduced anti-oxidant ability in the circulation and in the liver of offspring from diet-induced obese dams^11^. In particular, offspring developed hyperinsulinemia and fatty liver at 8 weeks of age, with no difference in body weight or adiposity with respect to controls: this finding is suggestive of differential programming of metabolic tissues leading to diabetes and fatty liver, which are well known obesity-related disorders^11^.

Another group of deregulated proteins that we identified in Ob-hA-MSCs is involved in the cytoskeleton and/or in cellular vesicle trafficking. In detail, ACTN1, several ANXs (ANX1, ANX2, ANX5, ANX6), LMNA and VIM were up-regulated in Ob-hA-MSCs vs Co-hA-MSCs. ACTN1 is a cytoskeletal protein that belongs to the superfamily of filamentous actin crosslinking proteins. It is a membrane-associated protein present in non-muscle cells, where it is concentrated in actin stress fiber ends and in adherens junctions^22^. ACTN1 plays a relevant role in cell motility; notably, in the immune response it drives cells to sites of inflammation^22^. ANXs are a family of Ca^+2^-regulated proteins: they are membrane-binding proteins that regulate the stabilization of membrane domains, membrane-cytoskeleton linking, and exocytic and endocytic events^23^. ANXA2 is an actin-binding protein involved in such cellular activities as the organization of membrane lipids at the site of actin cytoskeleton attachment, and whose activity correlates with inflammation. In fact, ANXA2 activates and stimulates the production of cytokines IL-6 and TNF-α as well as ICAM-1 production in macrophages *in vitro*^24^. Furthermore, in adipocytes, ANXA2 is a positive mediator of actin-dependent transport of GLUT4 vesicles on the membrane surface. In fact, ANXA2 inhibition in 3T3-L1 adipocytes resulted in reduction of GLUT4 translocation, whereas glucose-uptake and GLUT4 translocation were significantly higher in adipocytes transfected with wild-type ANXA2 than in cells transfected with control vector^25^. LMNA is a component of the nuclear lamina, which is the layer between chromatin and the inner nuclear membrane^26^. Accumulation of LMNA in hMSCs determines a premature aging phenotype that ultimately reduces the functionality of these cells^27^. LMNA accumulation in hMSCs deranges lipid metabolism and leads to structural and functional alterations of mitochondria and of the endoplasmic reticulum, which are organelles important for lipid homeostasis^28^. These alterations have been reported in such lipid-related metabolic diseases as obesity and type 2 diabetes^29^. Finally, VIM expression was enhanced in Ob-hA-MSCs. VIM is involved in fat droplet reorganization and its disruption during adipose differentiation of 3T3-L1 cells inhibits lipid droplet accumulation^30^; conversely, its increase could promote the lipid droplet accumulation in Ob-hA-MSCs. In line with the latter hypothesis, we previously observed an increased adipogenic potential in Ob-hA-MSCs with respect to Co-hA-MSCs^8^. Our previous finding of increased levels of ANXs, LMNA, VIM in visceral adipose tissue in morbidly obese subjects^31^ supports a link between these proteins and obesity. Lastly, levels of LMNA mRNA were found to be higher in the subcutaneous adipose tissue of obese subjects than in controls^32^.

The third group of proteins we identified were predicted to deregulate metabolic pathways being down- (ALDOC and ENO1) or up-expressed (PKM, PFKP and HK1) in Ob- vs Co-hA-MSCs. Protein ALDOC, by promoting the association of F-actin with GLUT4, was found to play a role in intracellular GLUT4 sequestration in subcutaneous adipose tissue^33^. It was hypothesized that ALDOC reduction could promote translocation of the protein GLUT4 in the membrane, thereby increasing glucose uptake and triglyceride synthesis and storage^33^. However, given the lack of experimental data, the role, if any, of these proteins in obesity or in obesity-associated disorders requires further investigation.

In conclusion, exposure to an obesogenic environment *in utero* is associated with proteome alterations in hA-MSCs. The proteins deranged are involved in the stress response, cytoskeleton and metabolic pathways, which are often associated with obesity-related phenotypes^10^,^11^,^25^,^28^. The possible role of these protein alterations in enhancing susceptibility to obesity or to obesity-related disorders in newborns of obese mothers requires further investigations. Notably, studies conducted in animal models of obesity during pregnancy^10^,^11^ indicate that cellular stress could trigger in human placenta metabolic alterations that predispose offspring to obesity-related disorders such as insulin resistance and fatty liver in fetal life and later in life. Our Ob-hA-MSC proteome data suggest that a similar mechanism could occur also in humans.

## Material and Methods

### Patients

Twelve obese (mean age 34.0 years) and 6 non-obese pregnant women (mean age 34.3 years) with pre-pregnancy BMI (mean/SD) 42.7/7.7 and 21.3/3.3 kg/m^2^, respectively, were enrolled at the Dipartimento di Neuroscienze e Scienze Riproduttive ed Odontostomatologiche, University of Naples Federico II. The exclusion criteria were neoplasia, viral infections, diabetes and metabolic syndrome. The clinical, anamnestic and family history, and biochemical data of each woman were recorded immediately before delivery. The birth weight, length, head circumference, 1 and 5 min Apgar indexes of infants were measured at delivery. All patients and controls gave their informed consent to the study. The study was performed according to the Helsinki II Declaration and was approved by the Ethics Committee of School of Medicine, University of Naples Federico II (authorization n. 248/08, 23/02/2009; amendment n. 248/08/ES1, 1/10/2014).

### Sample collection

Two maternal blood samples were collected from each enrolled woman immediately before delivery. One sample was used for DNA extraction, whereas the other was centrifuged and the serum was stored at −80 °C until required for the measurement of the main biochemical parameters and leptin/adiponectin concentrations by routine methods or by immunoassay (Bio-Rad, Hemel Hempstead, Herts, UK), respectively. The term placentas were collected at delivery by cesarean section. Placentas were processed immediately to isolate the hA-MSCs from the amniotic membranes, according to Parolini *et al.*^34^. The isolation, DNA typing (to confirm fetal origin) and immunophenotyping of hA-MSCs were performed as described elsewhere^8^.

### Two-Dimensional Fluorescence Difference Gel Electrophoresis

We pooled the hA-MSCs from two obese women to obtain a biological replicate (Ob-hA-MSCS). We performed a comparative experiment using six biological replicates of hA-MSCs from obese women and six biological replicates of hA-MSCs from non-obese women. DIGE experiments were performed as previously described^35^. The hA-MSCs were collected and resuspended in 0.5 ml of lysis buffer, containing 7M urea, 2M thiourea, 4% w/v chaps (3-[(3-cholamidopropyl)-dimethylammonium]-1-propane sulfonate), 30 mM Tris-HCl pH 7.5, cocktail of protease inhibitors (GE Healthcare, Piscataway, NJ). Cell debris was removed by centrifugation at 14,000 rpm at 4° C for 45 min. The cell lysate supernatant was precipitated using a 2D Clean up kit according to manufacturer’s instructions (GE Healthcare) and resuspended in 100 μL of lysis buffer.

The protein extract concentrations were determined and equal amounts of the protein lysates were labeled *in vitro* using two fluorescent cyanine minimal dyes (Cy3 and Cy5) that differed in excitation and emission wavelengths. A third cyanine dye (Cy2) was used to label a mixture of all samples as internal standard. The three differently labeled protein mixtures were pooled and subjected to isoelectric focusing through a non-linear pH range of 3–10 over a strip length of 24 cm. Strips were rehydrated without protein samples, with 450 μL of rehydration buffer containing 450 μL of DeStreak rehydration solution and 2% IPG Buffer pH 3−10 NL (GE Healthcare, Buckinghamshire, UK) overnight at room temperature. After rehydration, the strips were transferred to the Ettan IPGphor system (GE Healthcare) for isoelectric focusing. The samples were loaded on the strips with an equal volume of sample buffer containing 7M urea, 2M thiourea, 4% CHAPS, 0.2% DTT and 2% IPGphor buffer. The IPG strips were focused for 18 h at 20 °C as follows: 500 V for 4 h, linear gradient to 1000 V in 4 h, linear gradient to 10000 V in 4 h, step at 10000 V in 3 h, 300 V for 10 h.

The reducing and alkylating steps were performed between the first and the second electrophoretic step. Acrylamide strips were then transferred to the top of a classical SDS PAGE gel for a second orthogonal electrophoresis analysis. The Cy2, Cy3 and Cy5 images were obtained by scanning each of the four DIGE gels at an excitation/emission wavelength of 480/530 nm for Cy2, 520/590 nm for Cy3 and 620/680 nm for Cy5 using a Typhoon 9410 TM scanner (GE Healthcare). The semi-preparative gel was prepared according to the previously described procedure^35^, using 450 μg of protein from all replicates enrolled in this study. The semi-preparative gel was stained using GelCode™ Blue Stain Reagent (Thermo Fisher, Scientific, MA, USA) overnight. The semipreparative gel was scanned at 480/633 nm wavelengths using the Typhoon 9400 scanner (GE Healthcare). The images were analyzed with DeCyder software version 5.2 (GE Healthcare) in Batch Processing Mode. The maximum number of estimated spots per gel was fixed at 8000.

Detection and quantification of protein spots were carried out using the differential in-gel analysis (DIA), whereas protein spot matching among different gels was obtained using the biological variation analysis (BVA). The DIA module was used to compare Cy3/Cy5 image pairs with Cy2 internal standard from each gel. The DIA model was used also to detect spot boundaries and to calculate spot volume, normalized versus the volume of the corresponding spot present in the pool standard of the same gel. This analysis enables automated first level matching (“within-gel”) with a low experimental variation^36^. Image pairs were then matched between gels using the BVA feature. The results of the intra-gel comparison (DIA module) were imported into the BVA module. The Cy2 image containing the highest number of spots was designated the “master image” and used as template. The protein spots of the remaining internal standard images were automatically matched with the “master image”. Each spot intensity was expressed as a mean value of the 6 gels in order to reduce inter-gel variation. Spot intensities were then compared between the hA-MSCs from obese and from non-obese pregnant women. The Student’s *t-test* was used to determine the statistical significance of differences in spot intensity. Differentially regulated spots were defined as having a variation higher than 1.2 (P < 0.05) as reported elsewhere^37^. The accuracy of spots matching was verified, manually. The spot map containing spots differentially expressed in hA-MSCs from obese vs non-obese pregnant women were excised from the semi-preparative gel using the Ettan Spot Picker robotic system (GE Healthcare).

### Proteomic analysis

The spots of interest were excised, hydrolyzed and the peptide mixtures analyzed by mass spectrometry, MALDI-MS and LC-MS/MS using respectively 4800 Plus MALDI TOF/TOF™ Analyzer, Applied Biosystems 4800 Proteomics Analyzer (Applied Biosystems, Framingham, MA, USA) and a LC/MSD Trap XCT Ultra (Agilent Technologies, Palo Alto, CA, USA) equipped with a 1100 HPLC system and a chip cube (Agilent Technologies). MALDI spectra were acquired in the positive ion reflector mode using delayed extraction in the mass range between 800 and 4000 Da. LC-MSMS analysis was performed using data-dependent acquisition of one MS scan followed by MS/MS scans of the three most abundant ions in each MS scan. Raw data analyses were converted into a Mascot format text to identify proteins using Matrix Science software. The protein search considered the following parameters: non-redundant protein sequence database (NCBInr), specificity of the proteolytic enzyme used for the hydrolysis (trypsin), taxonomic category of the sample, no protein molecular weight was considered, up to one missed cleavage, cysteines as S-carbamidomethylcysteines, unmodified N- and C-terminal ends, methionines both unmodified and oxidized, putative pyro-Glu formation by Gln, precursor peptide maximum mass tolerance of 200 ppm, and a maximum fragment mass tolerance of 200 ppm.

### Western Blot Analysis

Protein extracts (30 μg) from Ob- and Co-hA-MSCs were analyzed by Western blot. Proteins were separated by 10% SDS-PAGE, and electroblotted onto nitrocellulose membranes (GE Healthcare). Nitrocellulose membranes were blocked with 5% Milk in PBS buffer with 0.1% v/v Tween (PBS-T), 20 for 2 h at room temperature. Immunoblotting was performed with the goat polyclonal antibody, anti-HSP90B1, anti-HSPB1 (Santa Cruz Biotechnology, Santa Cruz, CA, USA), rabbit polyclonal antibody anti-LMNA, anti-ENO1, anti-HSPA5 (Santa Cruz Biotechnology), anti-PKM (Adcam Discover More, Northampton, UK), mouse antibody anti-ANXA2, anti-ACTN1, anti-GAPDH (Santa Cruz Biotechnology). Human anti-ALDOC antibody (kindly donated by Prof. F. Salvatore, CEINGE-Biotecnologie Avanzate, Naples, Italy) was used as previously described^38^ to test ALDOC expression. Goat polyclonal antibodies were used at a 1:1000 dilution in milk/PBS-T 1% w/v, the rabbit polyclonal antibodies at a 1:1000 dilution, the mouse monoclonal antibodies at a 1:500 dilution, the anti-goat secondary antibody (Sigma Aldrich) at a 1:10000 dilution, and the anti-rabbit and anti-mouse secondary antibodies (GE Healthcare) at a 1:5000 dilution. Immunoblots were detected using HRP-conjugated secondary antibodies and enhanced chemiluminescence (GE Healthcare). The resulting Western blot images were scanned using Chemi Doc software (Biorad, Hercules, CA, USA) and analyzed using Quantity One software (Biorad). The optical density of each sample was measured and normalized using a GAPDH run on the same gel. The data are expressed as percent relative expression, namely, for each protein, the sample with the highest expression of that protein was set 100%.

### Statistical Analysis

The investigated parameters were expressed as mean ± standard deviation or mean/SEM. Student’s t test was used to compare group means, and a p-level < 0.05 was considered statistically significant. Statistical analyses were carried out with the PASW package for Windows (Ver.18; SPSS Inc. Headquarters, Chicago, Ill). In all cases, not-paired two-tailed Student’s t-tests were used to determine significance of the 2D-DIGE results, and false discovery rates were applied to all comparisons. We used the KEGG and DAVID bioinformatics tools to identify pathways containing altered proteins.

## Additional Information

**How to cite this article**: Capobianco, V. *et al.* Proteome analysis of human amniotic mesenchymal stem cells (hA-MSCs) reveals impaired antioxidant ability, cytoskeleton and metabolic functionality in maternal obesity. *Sci. Rep.* **6**, 25270; doi: 10.1038/srep25270 (2016).

## Supplementary Material

Supplementary Information

## Figures and Tables

**Table 1:** Identification and characterization of proteins differently expressed in Obese hA-MSCs vs Non-obese hA-MSCs.

Spot no.^a^	DIGE Obese/Non-obese hAMSCs^b^	DIGE (P-value)^c^	Protein name	Gene Symbol^d^	MW (Da)^e^	PI^f^	Gene ID^g^	Protein code^h^
3114	1.96	0.029	Annexin A5	ANXA5	35936	4.93	308	P08758
873	1.86	0.024	Alpha-actinin-1	ACTN1	103057	5.25	87	P12814
2710	1.77	0.031	Serpin B6	SERPINB6	42621	5.18	5269	P35237
2748	1.77	0.031	Annexin A1	ANXA1	38714	6.57	301	P04083
3711	1.66	0.0037	Ubiquitin carboxyl-terminal hydrolase isozyme L1	UCHL1	24824	5.33	7345	P09936
1536	1.63	0.026	Myosin-9	MYH9	226532	5.50	4627	P35579
			LIM domain and actin-binding protein 1	LIMA1	85225	6.41	51474	Q9UHB6
489	1.59	0.022	Filamin-A	FLNA	280739	5.70	2316	P21333
			Filamin-B	FLNB	278164	5.47	2317	O75369
662	1.49	0.00067	Heat shock protein 105 kDa	HSPH1	96864	5.27	10808	Q92598
3335	1.49	0.041	Prohibitin	PHB	29804	5.57	5245	P35232
660	1.47	0.0099	Programmed cell death 6-interacting protein	PDCD6IP	96023	6.13	10015	Q8WUM4
2879	1.47	0,0042	Elongation factor 1-delta	EEF1D	31121	4.90	1936	P29692
2730	1.47	0.049	Heterogeneous nuclear ribonucleoprotein A1	HNRNPA1	38746	9.17	3178	P09651
522	1.44	0.0018	Protein phosphatase 1 regulatory subunit 12A	PPP1R12A	115281	5.31	4659	O14974
1823	1.41	0.036	Dihydropyrimidinase-related protein 3	DPYSL3	61963	6.04	1809	Q14195
			Prelamin-A/C	LMNA	74139	6.57	4000	P02545
			T-complex protein 1 subunit zeta	CCT6A	58024	6.24	908	P40227
2813	1.40	0.022	Annexin A2	ANXA2	38604	7.57	302	P07355
			LIM and SH3 domain protein 1	LASP1	29717	6.61	3927	Q14847
521	1.39	0.0012	Protein phosphatase 1 regulatory subunit 12A	PPP1R12A	115281	5.31	4659	O14974
1047	1.38	0.0056	Myosin-9	MYH9	226532	5.50	4627	P35579
			Transitional endoplasmic reticulum ATPase	VCP	89265	5.14	7415	P55072
492	1.37	0.0056	Filamin-A	FLNA	280739	5.70	2316	P21333
			Filamin-B	FLNB	278164	5.47	2317	O75369
2925	1.37	0.053	Annexin A2	ANXA2	38604	7.57	302	P07355
			LIM and SH3 domain protein 1	LASP1	29717	6.61	3927	Q14847
574	1.33	0.040	Ribosome-binding protein 1	RRBP1	152472	8.69	6238	Q9P2E9
1996	1.32	0.049	Vimentin	VIM	53651	5.05	7431	P08670
452	1.28	0.0017	Nodal modulator 1	NOMO1	134324	5.54	23420	Q15155
918	1.27	6.4e–005	Ubiquitin-like modifier-activating enzyme 1	UBA1	117849	5.49	7317	P22314
			Alpha-actinin-1	ACTN1	103057	5.25	87	P12814
			Alanine-tRNA ligase, mitochondrial	AARS2	107340	5.87	57505	Q5JTZ9
1395	1.27	0.0018	ATP-dependent 6-phosphofructokinase, platelet type	PFKP	85596	7.50	5214	Q01813
			Protein transport protein Sec23A	SEC23A	86160	6.64	10484	Q15436
3408	1.26	0.0013	40S ribosomal protein SA	RPSA	32854	4.79	3921	P08865
1866	1.26	0.022	Pyruvate kinase PKM	PKM	57937	7.96	5315	P14618
2861	1.26	0.049	Annexin A2	ANXA2	38604	7.57	302	P07355
2955	1.25	0.038	LIM and SH3 domain protein 1	LASP1	29717	6.61	3927	Q14847
1476	1.23	0.0029	Stress-70 protein, mitochondrial	HSPA9	73680	5.87	3313	P38646
			Annexin A6	ANXA6	75873	5.41	309	P08133
604	1.23	0.0032	ATP-citrate syntase	ACLY	120839	6.95	47	P53396
			Leucine—tRNA ligase,cytoplasmic	LARS	134466	6.95	51520	Q9P2J5
279	1.23	0.014	Pyruvate kinase PKM	PKM	57937	7.96	5315	P14618
903	1.22	0.0011	Staphylococcal nuclease domain-containing protein 1	SND1	101996	6.74	27044	Q7KZF4
2769	1.22	0.016	Eukaryotic translation initiation factor 3, subunit I	EIF3I	36501	5.38	8668	Q13347
456	1.21	0.0005	Leucine-rich PPR motif-containing protein, mitochondrial	LRPRRC	157905	5.81	10128	P42704
1874	1.21	0.018	Pyruvate kinase PKM	PKM	57937	7.96	5315	P14618
1236	1.20	0.0079	LIM domainand actin-binding protein 1	LIMA1	85225	6.41	51474	Q9UHB6
1017	1.20	0.019	Programmed cell death 6-interacting protein	PDCD6IP	96023	6.13	10015	Q8WUM4
			Hexokinase-1	HK1	102485	6.36	3098	P19367
2770	−6.13	0.041	Annexin A1	ANXA1	38714	6.57	301	P04083
3162	−3.34	0.041	Chloride intracellular channel protein 1	CLIC1	26922	5.09	1192	O00299
3248	−2.21	0.051	S-methyl-5′-thioadenosine phosphorylase	MTAP	31236	6.75	4507	Q13126
1385	−2.05	0.019	78 kDa glucose-regulated protein	HSPA5 (Grp78)	72332	5.07	3309	P11021
3412	−1.93	0.0030	Heat shock protein beta-1	HSPB1 (HSP27)	22782	5.98	3315	P04792
3452	−1.70	0.00024	Heat shock protein beta-1	HSPB1 (HSP27)	22782	5.98	3315	P04792
3794	−1.59	0.031	Chain A, Three-Dimensional Structure Of Class Pi Glutathione S-Transferase From Human Placenta (Glutathione S-transferase P)	GSTP1	23355	5.43	2950	P09211
2345	−1.55	0.048	6-phosphogluconate dehydrogenase, decarboxylating	PGD	53139	6.8	5226	P52209
2606	−1.42	0.029	Fructose-bisphosphate aldolase C	ALDOC	39455	6.41	230	P09972
			Mitochondrial import receptor subunit TOM40 homolog	TOMM40	37893	6.79	10452	O96008
2326	−1.41	0.0093	Serpin H1	SERPINH1	46440	8.75	871	P50454
798	−1.39	0.046	Lon protease homolog, mitochondrial	LONP1	106489	6.01	9361	P36776
1243	−1.37	0.0038	Ran GTPase-activating protein 1	RANGAP1	63541	4.63	5905	P46060
2090	−1.36	0.041	Alpha-aminoadipic semialdehyde dehydrogenase	ALDH7A1	58487	8.21	501	P49419
			RuvB-like 1	RUVBL1	50228	6.02	8607	Q9Y265
2125	−1.35	0.017	Dihydrolipoyllysine-residue succinyltransferase component of 2-oxoglutarate dehydrogenase complex, mitochondrial	DLST	48755	9.10	1743	P36957
1245	−1.35	0.023	Caldesmon	CALD1	93231	5.62	800	Q05682
1310	−1.31	0.035	Protein disulfide-isomerase A4	PDIA4	72932	4.96	9601	P13667
			Acylamino-acid-relasing enzyme	APEH	81224	5.29	327	P13798
829	−1.30	0.028	Endoplasmin	HSP90B1 (Grp94)	92468	4.76	7184	P14625
1408	−1.28	0.00018	79 kDa glucose-regulated protein	HSPA5 (Grp78)	72332	5.07	3309	P11022
2258	−1.26	0.028	Alpha-enolase	ENO1	47168	7.01	2023	P06733
			6-phosphogluconate dehydrogenase, decarboxylating	PGD	53139	6.8	5226	P52209
1327	−1.23	0.014	Protein disulfide-isomerase A4	PDIA4	72932	4.96	9601	P13667
			Prolyl endopeptidase	PREP	80699	5.53	5550	P48147
2621	−1.23	0.049	Actin, cytoplasmic 2	ACTG1	41792	5.31	71	P63261
			COP9 signalosome complex subunit 4	COPS4	46268	5.57	51138	Q9BT78
1786	−1.22	0.0028	60 kDa heat shock protein, mitochondrial	HSPD1 (HSP60)	61054	5.70	3329	P10809
1377	−1.21	0.049	78 kDa glucose-regulated protein	HSPA5 (Grp78)	72332	5.07	3309	P11021
			Heat shock cognate 71 kDa protein	HSPA8	70898	5.37	3312	P11142

^a^Spot numbering to indicate the positions of spots in preparative gel.

^b^Average abundance ratio (Obese/Non-obese hA-MSCs) as calculated by DeCyder Analysis.

^c^P-value at Student’s *t* test.

^d^Gene symbol from NCBI.

^e^Theoretical molecular weight (Da).

^f^Theoretical pI.

^g^Gene ID from NCBI.

^h^Protein code from Swiss Prot.
