# Supplementary material for: Proteome analysis of human amniotic mesenchymal stem cells (hA-MSCs) reveals impaired antioxidant ability, cytoskeleton and metabolic functionality in maternal obesity
Source: Sci Rep. 2016 Apr 29;6:25270. doi: 10.1038/srep25270 (PMC4850482; doi:10.1038/srep25270)
Supplement: Supplementary Information [file srep25270-s1.pdf]

Supplementary Information for

Proteome analysis of human amniotic mesenchymal stem cells (hA-MSCs) reveals impaired antioxidant ability, cytoskeleton and metabolic functionality in maternal obesity

Valentina Capobianco[1*], Marianna Caterino[1,2*], Laura Iaffaldano[1], Carmela Nardelli[1,2], Angelo Sirico[3], Luigi Del Vecchio[1,2], Pasquale Martinelli[3], Lucio Pastore[1,2], Pietro Pucci[1,4], Lucia Sacchetti[1]†

[1]CEINGE-Biotecnologie Avanzate S.C.a R.L., Via Gaetano Salvatore 486, 80145 Naples, Italy

[2]Dipartimento di Medicina Molecolare e Biotecnologie Mediche, Università degli Studi di Napoli Federico II, Via S. Pansini 5, 80131 Naples, Italy

[3]Dipartimento di Neuroscienze e Scienze Riproduttive ed Odontostomatologiche, Via S. Pansini 5, 80131 Naples, Italy.

[4]Dipartimento di Scienze chimiche, Via Cintia, Complesso Monte Sant'Angelo 21, 80126 Naples, Italy

*These authors contributed equally to the paper

†Corresponding author:

Lucia Sacchetti, e-mail: sacchett@unina.it

This file include:

1. Supplementary Table S1
2. Supplementary Table S2
3. Supplementary Figure S1
4. Supplementary Figure S2

(a)

**Pregnant women features**	**Obese**	**Non-obese**
Subjects (n)	12	6
Age (years)	34.0 ± 3.5	34.3 ± 6.8
Weight (kg)[a]	111.3 ± 19.9	59.8 ± 5.1
Height (m)	1.60 ± 0.1	1.67 ± 2.2
BMI pre-pregnancy (kg/m^2)[a]	42.7 ± 7.7	21.3 ± 3.3
Weight gain in pregnancy	9.5 ± 5.2	14.0 ± 4.2
Systolic blood pressure (mmHg)	122.7 ±12.9	120.0 ± 0.5
Diastolic blood pressure (mmHg)	79.0 ± 9.7	76.7 ± 5.7
Cardiac frequency	80.5 ± 4.4	82.5 ± 3.5
Gestational age	39.0 ± 1.2	38.3 ± 0.5
Glucose (mmol/L)	4.3 ± 0.6	3.8 ± 0.4
Cholesterol (mmol/L)	7.1 ± 2.1	7.4 ± 0.5
Triglycerides (mmol/L)	2.8 ± 1.1	2.2 ± 1.3
AST (U/L)	15.1 ± 4.7	19.7 ± 8.1
ALT (U/L)	13.3 ± 4.4	14.7 ± 13.8
ALP (U/L)	131.7 ± 46.0	126.2 ± 39.0
GGT (U/L)	10.7 ± 5.8	9.5 ± 5.7
PCR (mg/L)	6.4 ± 6.1	6.4 ± 2.6
Albumin (g/dl)	3.2 ± 0.4	3.4 ± 0.3
Total proteins (g/dl)	6.1 ± 0.6	6.1 ± 0.4
Leptin (L) (ng/ml)[b]	35.0 ± 8.5	12.3 ± 7.9
Adiponectin (A) (µg/ml)	7.2 ± 4.3	10.8 ± 2.8
L/A[c]	7.4 ± 3.6	1.2 ± 0.7

(b)

**Newborn features**	**Obese newborns**	**Non-obese newborns**
Birth weight (g)	3416.3 ± 502.7	3212.5 ± 502.2
Length (cm)	50.3 ± 1.3	50.7 ± 2.2
Head circumference (cm)	34.8 ± 1.4	35.0 ± 0.5
Apgar 1’	7.6 ± 1.0	8.0 ± 0.8
Apgar 5’	8.8 ± 0.5	8.7 ± 0.5

Supplementary Table S1. Clinical and biochemical characteristics of obese and non-obese pregnant women (**a**) and of their newborns (**b**) Data are expressed as mean ± Standard Deviation. Statistically significant difference at the Student’s *t* test. $^{a}P < 0.0001$; $^{b}P = 0.002$; $^{c}P = 0.01$

(a) **Pathways predicted by DAVID**

**Annotation Cluster 1**	**Enrichment Score: 4.62**	**Proteins**	**P_Value**
GOTERM_BP_FAT	response to unfolded protein	HSPH1, VCP, HSPB1, CLIC1, HSPD1, SERPINH1, HSPA8	3.2E-7
SP_PIR_KEYWORDS	stress response	HSPH1, HSPB1, HSPD1, SERPINH1, HSPA8, HSPA9	1.8E-6
GOTERM_BP_FAT	response to protein stimulus	HSPH1, VCP, HSPB1, CLIC1, HSPD1, SERPINH1, HSPA8	3.6E-6
SP_PIR_KEYWORDS	heat shock	HSPB1, HSPD1, SERPINH1	5.5E-5
SP_PIR_KEYWORDS	stress-induced protein	HSPB1, HSPD1, SERPINH1	2.6E-4
GOTERM_BP_FAT	response to organic substance	HSPH1, VCP, ALDOC, HSPB1, CLIC1, HSPD1, ANXA5, SERPINH1, HSPA8	6.6E-3

**Annotation Cluster 2**	**Enrichment Score: 4.46**	**Proteins**	**P_Value**
GOTERM_CC_FAT	pigment granule	ANXA6, HSP90B1, SND1, PDCD6IP, PDIA4, HSPA5, HSPA8, ANXA2	5.6E-8
GOTERM_CC_FAT	melanosome	ANXA6, HSP90B1, SND1, PDCD6IP, PDIA4, HSPA5, HSPA8, ANXA2	5.6E-8
GOTERM_CC_FAT	cytoplasmic membrane-bounded vesicle	ANXA6, SEC23A, HSP90B1, SND1, ACTN1, PDCD6IP, PDIA4, HSPA5, HSPD1, HSPA8, ANXA2	5.2E-5
GOTERM_CC_FAT	membrane-bounded vesicle	ANXA6, SEC23A, HSP90B1, SND1, ACTN1, PDCD6IP, PDIA4, HSPA5, HSPD1, HSPA8, ANXA2	6.8E-5
GOTERM_CC_FAT	cytoplasmic vesicle	ANXA6, SEC23A, HSP90B1, SND1, ACTN1, PDCD6IP, PDIA4, HSPA5, HSPD1, HSPA8, ANXA2	1.9E-4
GOTERM_CC_FAT	vesicle	ANXA6, SEC23A, HSP90B1, SND1, ACTN1, PDCD6IP, PDIA4, HSPA5, HSPD1, HSPA8, ANXA2	2.7E-4

**Annotation Cluster 3**	**Enrichment Score: 4.33**	**Proteins**	**P_Value**
SP_PIR_KEYWORDS	molecular chaperone	LONP1, CCT6A, HSPD1, SERPINH1, HSPA8	5.6E-8
SP_PIR_KEYWORDS	stress response	HSPH1, HSPB1, HSPD1, SERPINH1, HSPA8, HSPA9	1.8E-6
INTERPRO	Heat shock protein Hsp70	HSPH1, HSPA5, HSPA8, HSPA9	9.1E-6
INTERPRO	Heat shock protein 70	HSPH1, HSPA5, HSPA8, HSPA9	9.1E-6
GOTERM_MF_FAT	unfolded protein binding	HSP90B1, CCT6A, HSPA5, HSPD1, SERPINH1, HSPA8, HSPA9	1.1E-5
INTERPRO	Heat shock protein 70, conserved site	HSPH1, HSPA5, HSPA8, HSPA9	1.9E-5
SP_PIR_KEYWORDS	Chaperone	HSP90B1, CCT6A, HSPD1, SERPINH1, HSPA8, HSPA9	1.2E-4
PIR_SUPERFAMILY	PIRSF002581:chaperone HSP70	HSPA5, HSPA8, HSPA9	9.9E-4
GOTERM_BP_FAT	protein folding	HSP90B1, CCT6A, HSPD1, HSPA8, HSPA9	5.0E-3
GOTERM_CC_FAT	cell surface	HSPB1, HSPA5, HSPD1, HSPA8, HSPA9	5.0E-2

**Annotation Cluster 4**	**Enrichment Score: 3.76**	**Proteins**	**P_Value**
SP_PIR_KEYWORDS	molecular chaperone	LONP1, CCT6A, HSPD1, SERPINH1, HSPA8	5.6E-8
SP_PIR_KEYWORDS	ATP	LONP1, PFKP, HK1, HSPD1, HSPA8	5.9E-3

GOTERM_MF_FAT	ATPase activity	LONP1, VCP, RUVBL1, HSPD1, MYH9, HSPA8	1.5E-2

**Annotation Cluster 5**	**Enrichment Score: 3.68**	**Proteins**	**P_Value**
SP_PIR_KEYWORDS	atp-binding	PFKP, HK1, ACLY, CCT6A, MYH9, ACTG1, HSPH1, LONP1, HSP90B1, VCP, UBA1, LARS, HSPA5, HSPD1, RUVBL1, AARS2, HSPA8, HSPA9	2.8E-7
SP_PIR_KEYWORDS	nucleotide-binding	PFKP, HK1, ACLY, CCT6A, MYH9, ACTG1, HSPH1, LONP1, HSP90B1, VCP, UBA1, LARS, HSPA5, HSPD1, RUVBL1, AARS2, HSPA8, HSPA9	7.7E-6
GOTERM_MF_FAT	ATP binding	PFKP, HK1, ACLY, CCT6A, MYH9, ACTG1, HSPH1, LONP1, HSP90B1, VCP, UBA1, LARS, HSPA5, HSPD1, RUVBL1, AARS2, HSPA8, HSPA9	1.3E-4
GOTERM_MF_FAT	adenyl ribonucleotide binding	PFKP, HK1, ACLY, CCT6A, MYH9, ACTG1, HSPH1, LONP1, HSP90B1, VCP, UBA1, LARS, HSPA5, HSPD1, RUVBL1, AARS2, HSPA8, HSPA9	1.6E-4
GOTERM_MF_FAT	adenyl nucleotide binding	PFKP, HK1, ACLY, CCT6A, MYH9, ACTG1, HSPH1, LONP1, HSP90B1, VCP, UBA1, LARS, HSPA5, HSPD1, RUVBL1, AARS2, HSPA8, HSPA9	2.9E-4
GOTERM_MF_FAT	purine nucleoside binding	PFKP, HK1, ACLY, CCT6A, MYH9, ACTG1, HSPH1, LONP1, HSP90B1, VCP, UBA1, LARS, HSPA5, HSPD1, RUVBL1, AARS2, HSPA8, HSPA9	3.5E-4
GOTERM_MF_FAT	nucleoside binding	PFKP, HK1, ACLY, CCT6A, MYH9, ACTG1, HSPH1, LONP1, HSP90B1, VCP, UBA1, LARS, HSPA5, HSPD1, RUVBL1, AARS2, HSPA8, HSPA9	3.8E-4
GOTERM_MF_FAT	purine ribonucleotide binding	PFKP, HK1, ACLY, CCT6A, MYH9, ACTG1, HSPH1, LONP1, HSP90B1, VCP, UBA1, LARS, HSPA5, HSPD1, RUVBL1, AARS2, HSPA8, HSPA9	1.7E-3
GOTERM_MF_FAT	ribonucleotide binding	PFKP, HK1, ACLY, CCT6A, MYH9, ACTG1, HSPH1, LONP1, HSP90B1, VCP, UBA1, LARS, HSPA5, HSPD1, RUVBL1, AARS2, HSPA8, HSPA9	1.7E-3
GOTERM_MF_FAT	nucleotide binding	PGD, PFKP, HK1, ACLY, CCT6A, MYH9, HNRNPA1, ACTG1, HSPH1, LONP1, HSP90B1, VCP, UBA1, LARS, HSPA5, HSPD1, RUVBL1, AARS2, HSPA8, HSPA9	2.4E-3
GOTERM_MF_FAT	purine nucleotide binding	PFKP, HK1, ACLY, CCT6A, MYH9, ACTG1, HSPH1, LONP1, HSP90B1, VCP, UBA1, LARS, HSPA5, HSPD1, RUVBL1, AARS2, HSPA8, HSPA9	2.8E-3

**Annotation Cluster 6**	**Enrichment Score: 3.66**	**Proteins**	**P_Value**
SP_PIR_KEYWORDS	cytoskeleton	ACTG1, LIMA1, LASP1, CALD1, HSPB1, ACTN1, RANGAP1, RUVBL1, PDCD6IP, FLNB, FLNA	2.1E-5
GOTERM_CC_FAT	intracellular non-membrane-bounded organelle	RPSA, LIMA1, RRBP1, CALD1, ALDOC, VIM, UCHL1, LMNA, ANXA1, ACTN1, RANGAP1, DPYSL3, MYH9, FLNB, HNRNPA1, FLNA, ACTG1, LONP1, VCP, LASP1, HSPB1, PDCD6IP, RUVBL1, HSPA9	5.5E-5
GOTERM_CC_FAT	non-membrane-bounded organelle	RPSA, LIMA1, RRBP1, CALD1, ALDOC, VIM, UCHL1, LMNA, ANXA1, ACTN1, RANGAP1, DPYSL3, MYH9, FLNB, HNRNPA1, FLNA, ACTG1, LONP1, VCP, LASP1, HSPB1, PDCD6IP, RUVBL1, HSPA9	5.5E-5
GOTERM_CC_FAT	cytoskeleton	LIMA1, ALDOC, CALD1, VIM, LMNA, ANXA1, ACTN1, DPYSL3, MYH9, FLNB, FLNA, ACTG1, LASP1, HSPB1, PDCD6IP, RUVBL1	2.2E-4

GOTERM_CC_FAT	cytoskeletal part	LIMA1, LASP1, CALD1, VIM, LMNA, HSPB1, RUVBL1, PDCD6IP, MYH9	3.4E-2

**Annotation Cluster 7**	**Enrichment Score: 3.64**	**Proteins**	**P_Value**
SP_PIR_KEYWORDS	acetylated amino end	ANXA6, ACTG1, APEH, PGD, ANXA1, ANXA5, HNRNPA1, ANXA2	2.3E-8
SP_PIR_KEYWORDS	duplication	ANXA6, ANXA1, PFKP, HK1, ACTN1, PDIA4, ANXA5, FLNA, ANXA2	4.5E-7
SMART	SM00335:ANX	ANXA6, ANXA1, ANXA5, ANXA2	4.4E-6
SP_PIR_KEYWORDS	endonexin fold	ANXA6, ANXA1, ANXA5, ANXA2	4.4E-6
SP_PIR_KEYWORDS	annexin	ANXA6, ANXA1, ANXA5, ANXA2	9.7E-6
UP_SEQ_FEATURE	repeat:Annexin 1	ANXA6, ANXA1, ANXA5, ANXA2	9.9E-6
UP_SEQ_FEATURE	repeat:Annexin 2	ANXA6, ANXA1, ANXA5, ANXA2	9.9E-6
UP_SEQ_FEATURE	repeat:Annexin 3	ANXA6, ANXA1, ANXA5, ANXA2	9.9E-6
UP_SEQ_FEATURE	repeat:Annexin 4	ANXA6, ANXA1, ANXA5, ANXA2	9.9E-6
SP_PIR_KEYWORDS	calcium/phospholipid-binding	ANXA6, ANXA1, ANXA5, ANXA2	1.2E-5
INTERPRO	IPR001464:Annexin	ANXA6, ANXA1, ANXA5, ANXA2	1.5E-5
INTERPRO	IPR018252:Annexin repeat, conserved site	ANXA6, ANXA1, ANXA5, ANXA2	1.5E-5
INTERPRO	IPR018502:Annexin repeat	ANXA6, ANXA1, ANXA5, ANXA2	1.5E-5
SP_PIR_KEYWORDS	calcium binding	ANXA6, HSP90B1, ANXA1, ACTN1, ANXA5, ANXA2	1.6E-5
SP_PIR_KEYWORDS	phospholipid binding	ANXA6, ANXA1, ANXA5, ANXA2	4.0E-5
PIR_SUPERFAMILY	PIRSF002359:annexin I	ANXA6, ANXA1, ANXA5, ANXA2	4.5E-5
GOTERM_MF_FAT	GO:0005544~calcium-dependent phospholipid binding	ANXA6, ANXA1, ANXA5, ANXA2	1.9E-4
GOTERM_MF_FAT	GO:0004859~phospholipase inhibitor activity	ANXA1, ANXA5, ANXA2	1.2E-3
GOTERM_MF_FAT	GO:0055102~lipase inhibitor activity	ANXA1, ANXA5, ANXA2	1.9E-3
GOTERM_MF_FAT	GO:0004857~enzyme inhibitor activity	SERPINB6, ANXA1, HSPA5, ANXA5, SERPINH1, ANXA2	6.5E-3
GOTERM_MF_FAT	GO:0005543~phospholipid binding	ANXA6, ANXA1, ANXA5, ANXA2	4.3E-2

(b) Pathways predicted by KEGG

**Protein processing in endoplasmic reticulum P=7.27e-11; adjP=2.33e-09**	
**Gene Symbol**	**Gene Name**
SEC23A	Sec23 homolog A (S. cerevisiae)
HSPH1	heat shock 105kDa/110kDa protein 1
HSP90B1	heat shock protein 90kDa beta (Grp94), member 1
HSPA5	heat shock 70kDa protein 5 (glucose-regulated protein, 78kDa)
HSPA8	heat shock 70kDa protein 8
RRBP1	ribosome binding protein 1 homolog 180kDa (dog)
VCP	valosin containing protein

PDIA4	protein disulfide isomerase family A, member 4

**Glycolysis / Gluconeogenesis P=3.91e-10; adjP=6.26e-09**	
**Gene Symbol**	**Gene Name**
ALDH7A1	aldehyde dehydrogenase 7 family, member A1
HK1	hexokinase 1
PKM	pyruvate kinase, muscle
ENO1	enolase 1, (alpha)
ALDOC	aldolase C, fructose-bisphosphate
PFKP	phosphofructokinase, platelet

**Metabolic pathways P=2.87e-06; adjP=3.06e-05**	
**Gene Symbol**	**Gene Name**
ALDOC	aldolase C, fructose-bisphosphate
ACLY	ATP citrate lyase
ALDH7A1	aldehyde dehydrogenase 7 family, member A1
HK1	hexokinase 1
PKM	pyruvate kinase, muscle
ENO1	enolase 1, (alpha)
DLST	dihydrolipoamide S-succinyltransferase
PFKP	phosphofructokinase, platelet
MTAP	methylthioadenosine phosphorylase
PGD	phosphogluconate dehydrogenase

**Focal adhesion P=8.33e-06; adjP=5.33e-05**	
**Gene Symbol**	**Gene Name**
ACTN1	actinin, alpha 1
FLNA	filamin A, alpha
PPP1R12A	protein phosphatase 1, regulatory subunit 12A
ACTG1	actin, gamma 1
FLNB	filamin B, beta

**Pentose phosphate pathway P=6.95e-06; adjP=5.33e-05**	
**Gene Symbol**	**Gene Name**
ALDOC	aldolase C, fructose-bisphosphate

PFKP	phosphofructokinase, platelet
PGD	phosphogluconate dehydrogenase

**Fructose and mannose P=1.68e-05; adjP=8.96e-05**	
**Gene Symbol**	**Gene Name**
HK1	hexokinase 1
ALDOC	aldolase C, fructose-bisphosphate
PFKP	phosphofructokinase, platelet

**Arrhythmogenic right ventricular cardiomyopathy (ARVC) P=0.0001; adjP=0.0004**	
**Gene Symbol**	**Gene Name**
ACTN1	actinin, alpha 1
ACTG1	actin, gamma 1
LMNA	lamin A/C

**RNA degradation P=0.0001; adjP=0.0004**	
**Gene Symbol**	**Gene Name**
HSPA9	heat shock 70kDa protein 9 (mortalin)
HSPD1	heat shock 60kDa protein 1 (chaperonin)
ENO1	enolase 1, (alpha)

**Regulation of actin cytoskeleton P=0.0002; adjP=0.0007**	
**Gene Symbol**	**Gene Name**
MYH9	myosin, heavy chain 9, non-muscle
ACTN1	actinin, alpha 1
PPP1R12A	protein phosphatase 1, regulatory subunit 12A
ANXA2	annexin 2
ACTG1	actin, gamma 1

Supplementary Table S2. List of the significantly impaired pathways in Ob-hA-MSCs respect to Co-hA-MSCs, and proteins included in each of them as predicted by DAVID (a) and KEGG (b).

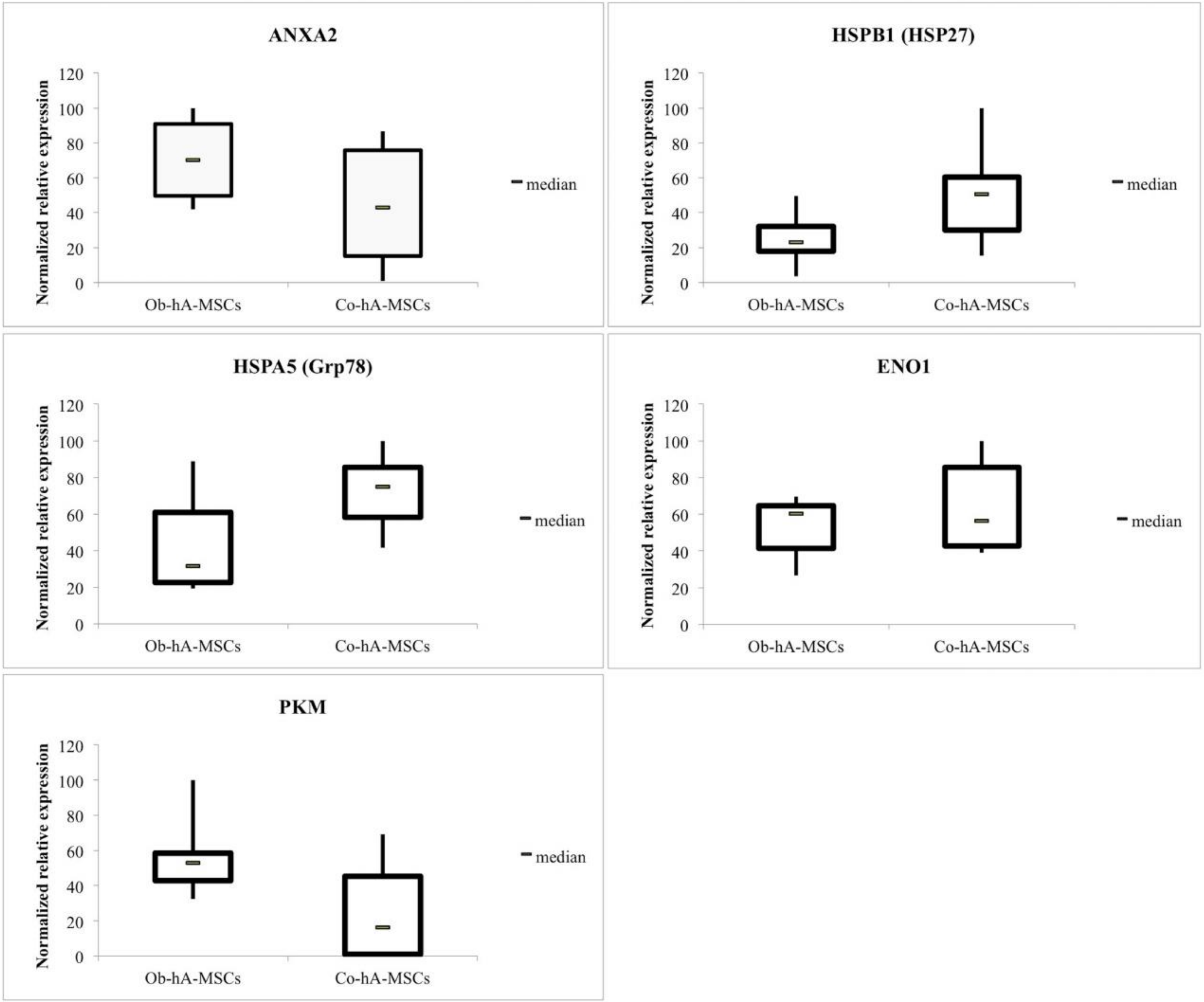

Supplementary Figure S1. Western blot evaluation of differently expressed proteins in Ob-hA-MSCs vs Co-hA-MSCs. The protein expression quantification for the selected proteins (ANXA2, HSPB1, HSPA5, ENO1 and PKM) normalized to GAPDH. The data are expressed as percent relative expression, i.e. for each protein, the sample with the highest expression of that protein was set 100%. The bottom and top of each box represent the 25th and 75th percentile, respectively; the thick band inside each box shows the 50th percentile (the median). The ends of the whiskers represent the minimum and maximum values of each group of data.

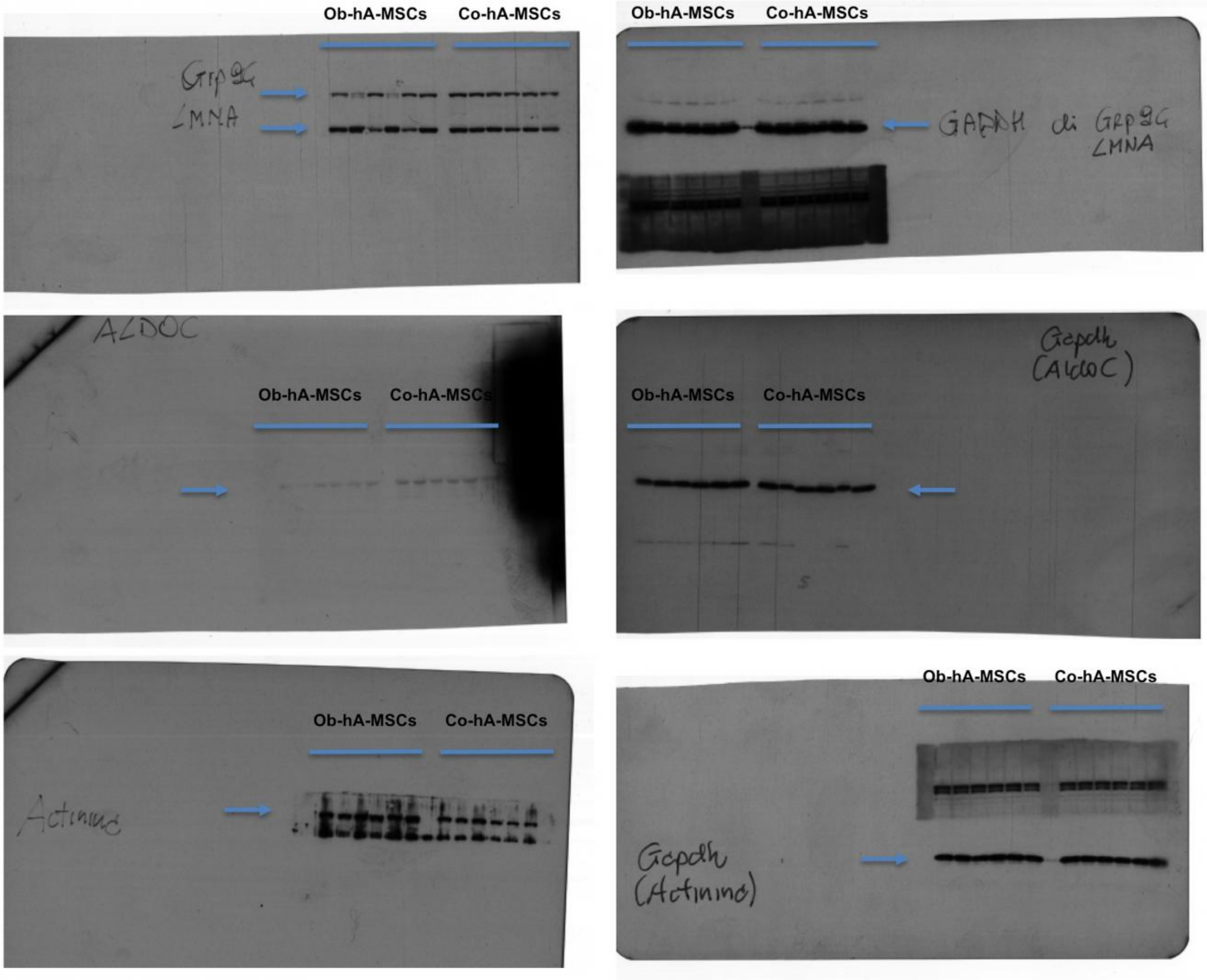

Supplementary Figure S2. Full-length blots of statistically significant proteins [HSP90B1 (Grp94), LMNA, ACTN1 and ALDOC] in Ob-hA-MSCs vs Co-hA-MSCs.
